# Analysis of Hepatitis E virus (HEV) X-domain structural model

**DOI:** 10.6026/97320630014398

**Published:** 2018-07-31

**Authors:** Thakur Vikram, Pradeep Kumar

**Affiliations:** 1 Department of Virology, Postgraduate Institute of Medical Education and Research (PGIMER), Sec-12, Chandigarh, India; 2 Faculty of Applied Sciences and Biotechnology, Shoolini University, Solan, (HP) India

**Keywords:** Hepatitis E Virus, ORF1, X domain model, Ligand binding site

## Abstract

Hepatitis E viral infection is now emerging as a global health concern, which needs to be addressed. Mechanism of viral replication and release is attributed by the different genomic component of HEV. However, few proteins/domain like X and Y domain remain unexplored, so we aim to explore the physiochemical, structural and functional features of HEV ORF-1 X domain. Molecular modeling of the unknown X domain was carried out using Phyre2 and Swiss Model. Active ligand binding sites were predicted using Phyre2. The X-domain protein found to be stable and acidic in nature with high thermostability and better hydrophilic property. Twelve binding sites were predicted along with putative transferase and catalytic functional activity. Homology modeling showed 10 binding sites along with Mg2+ and Zn2+ as metallic heterogen ligands binding to predicted ligand-binding sites. This study may help to decipher the role of this unexplored X-domain of HEV, thereby improving our understanding of the pathogenesis of HEV infection.

## Background

Hepatitis E virus (HEV) is recently evolving as a global emerging disease with neurological, haematological manifestations in addition to acute and chronic liver infection [1, 2]. Widely accounts for the 20-30% mortality in the HEV infected pregnant ladies in their third trimester [3], recent evidences of HEV in solid organ transplant patients, blood donors, and incidence of vertical transmission to newborns with severe maternal and fetal outcome, obviates the need to explore in depth the virus itself. Even the recent reports of the ribavirin resistance in HEV are alarming, as there is no effective FDA approved vaccine against HEV [4].

HEV, a small (~32nm), non-enveloped, single stranded (+) sense RNA virus is the main aetiological agent of Hepatitis E infection [5]. On the account of variations in open reading frame 2 (ORF-2), HEV recognised with eight genotypes and a common single serotype [6]. The ~7.2 Kb genome comprised of three ORFs, (ORF1, ORF2, and ORF3) with 5' (methylguanine-caped) and 3' (polyadenylated) non-coding terminal regions. ORF2 codes for the structural capsid protein [7], whereas virus infectivity and release is modulated by ORF3 phosphoprotein [8]. ORF1 codes for a polyprotein [9], which process to seven functional and/or putative domains viz. putative methyltransferase (MT), Ydomain (Y), papain-like cysteine protease (PCP), proline-rich hyper variable region (PRR/HVR), X-domain (X), helicase (Hel) and RNA-dependent RNA-polymerase (RdRp). Interestingly, few researchers studied the role of MT, PRR/HVR, Hel and RdRp [10, 11, 12, 13] in viral replication using molecular and biochemical characterization.

Recently, molecular study by Parvez MK, 2017 [14] suggested the role of Y domain sequence (a.a 239-439) in HEV life cycle through gene regulation and/or ER membrane binding in replication complexes. Allen et al 2003 [15], classify X domain to ADP-ribose- 1' monophosphate of macro-domain protein family. Although there is lack of significant sequence homology of viral X domains with phosphatases, yet some viruses are shown to have Appr-1- pase activity [16, 17], due to common macro-domain fold (Asparagine-rich (Asn) catalytic site).

So far, HEV ORF1X domain is known to interact with cellular ADP-ribose protein (involved in host pathogenesis), as a putative IFN antagonist in HEV replicating hepatoma cell [17, 18]. Also a potential Appr-1-pase active site (N806/N809/H812/G815-817) (Aspartate (Asp)/His/Glycine) was predicted in HEV X domain, HEV Appr-1-pase formed predicted β3-α2 secondary structure and X domain C terminal interact directly with MT and ORF3 through I66-67/I101-102 residues [19, 20, 21].

However, structure of this X domain is not reported yet. Also the detailed physiochemical characterization and putative structure with ligand binding active sites is not elucidated, so we proposed an in-silico 3-D structure prediction of HEV X domain using homology modelling.

## Methodology

### Retrieval of the target (X-Domain) amino acid sequence

The amino acid sequence of X-Domain (HEV ORF 1) protein was obtained from NCBI sequence database (http://www.ncbi.nlm.nih.gov/polyprotein/NP_056779.1). The main source of the sequence with gene ID-1494415 of HEVgp01 ORF-1; Seq: NC_001434.1 (4 … 5085). Polyprotein NP_056779.1; protein: protein structural polyprotein pORF1; Gene- ORF1; Organism: Hep E virus genotype 1 (Isolate Human/China/HeBei/1987(HEV); UniProtKB: >sp/Q81862/785-942;Pfam (X-Protein/Domain: 785-942). Due to unavailability of 3-D structure in PDB, modelling of this unexplored domain was undertaken utilizing 158 a. a. long sequence of X domain.

### Physiochemical characterization

Physiochemical properties of the retrieved sequence were determined using two web-based servers. ProtParam tool (Expasy) (http://us.expasy.org/tools/protparam.html) employed for the prediction of amino acid composition, instability and aliphatic indice, extinction coefficients and grand average of hydropathicity (GRAVY) [22]. Theoretical isoelectrical point (pI) was calculated using Sequence Manipulation Suite (SMS) Version 2 (http://www.bioinformatics.org/sms2/proteiniep.html).

### Secondary structure prediction of HEV X-domain protein

The self-optimized prediction method with alignment (SOPMA) software [23] and PSIPRED program (http://bioinf.cs.ucl.ac.uk/psipred) was used to predict the secondary structure of X Domain protein (target). Disorder prediction was performed using DISOPRED tool. Predict Protein software (https://predictprotein.org) including PROFsecwas also used to predict secondary structure [24].

### Protein binding sites and Gene ontology prediction of Xdomain

Protein-protein binding sites were predicted by profISIS [25] by identifying interacting residues from sequence alone by combining predicted structural features with evolutionary information. Molecular, cellular and biological functions were predicted by a Gene Ontology (GO) prediction method Metastudent [26] via homology to known annotated proteins.

### Homology modelling and validation of X-domain

There is no experimentally deduced 3D structure available for X domain protein in protein data bank (PDB), therefore homology modelling of the protein (X domain) was done using two program Swiss Model and Phyre2 (http://www.sbg.bio.ic.ac.uk/phyre2) [27, 28]. Secondary structure has also been predicted using Phyre2. 3D model of X domain generated from Swiss-Model and Phyre2 was compared and only the most suitable 3D model was selected for final validation. The final modelled structure was validated using Ramachandran plot analysis (PROCHECK) (http://nihserver.mbi.ucla.edu/SAVES) for sterio-chemical property. The final predicted model was submitted to the 3D LigandSite [29] server to predict the potential binding site.

## Result and Discussion

### Physiochemical characterization of X Domain

The amino acid sequence of HEV X domain was retrieved in FASTA format andused as query sequence for determination of physiochemical parameters. The instability index of 37.18 (<40) indicated the stable nature of X domain protein [30]. The protein is acidic in nature (pI 5.94, 6.34) with molecular weight of the 17.43kDa. High extinction coefficient values (28670) indicate the presence of Cys, Trp and Tyr residues [31]. Higher aliphatic index values (70.57) of the query protein suggested as a positive factor for increased thermos-stability for a wide temperature range [32]. Hydrophilic nature of the protein and the possibility of better interaction with water [33] were indicated by the lower grand average of hydropathicity GRAVY indices value (-0.278) as shown in Table 1.

### Secondary structure prediction

**Figure 1:**
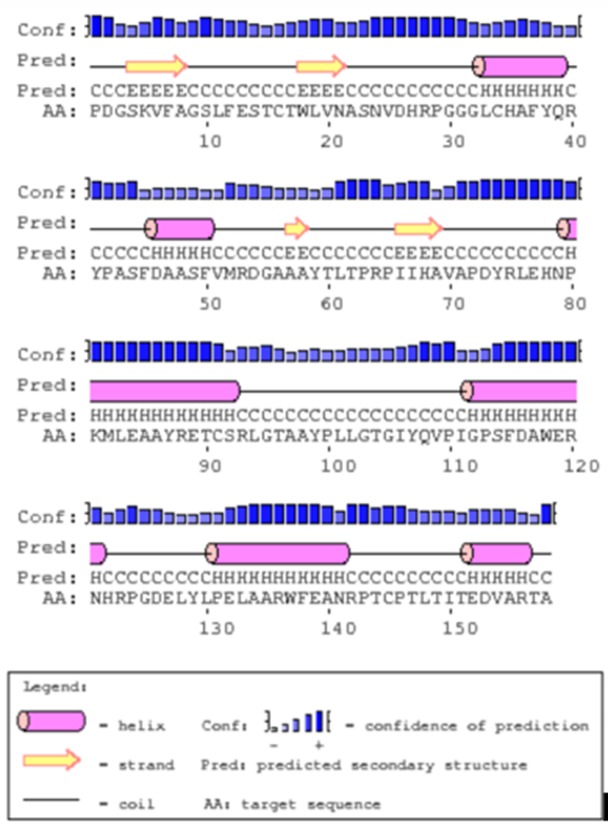
Secondary structure prediction by PSIPRED

The default parameters (similarity threshold: 8; window width: 17) were considered by SOPMA for the secondary structure prediction with >70% prediction accuracy. Utilising 511 proteins (sub-database) and 15 aligned proteins, SOPMA predicted 40.51% of residues as random coils in comparison to Alpha helix (34.81%), extended strand (20.25%) and Beta turn (4.43%) as shown in Table 2. PSIPRED showing the higher confidence of prediction of helix, strand and coil (Figure 1). Secondary structure prediction by PROFsec (PredictProtein) employing neural network system, provide the prediction accuracy of more than 72%.

42.41% helix confirmation (α; ∏; 3_10-helix), 44.30% loop (L) followed by 13.29% beta strand (E=extended strand in beta sheet conformation) was predicted in X domain. Intrinsic disorder profile was computed using DISOPRED and >90% of the amino acid are below the confidence score of 0.5 for disordered condition, suggested the lowest possibility of distortion and conferred the high stability to the predicted protein.

### Protein binding sites and Gene ontology prediction

Binding sites were predicted using predict protein software (profISIS), where 12 different protein binding sites were identified at positions viz.: 28-30; 46-47; 49; 59-60; 73-78; 88-89; 93; 108; 128; 131-133; 135; 141 (data not shown). Gene ontology predicted and categories the functional aspects as cellular, molecular and biological, where this X domain protein found to be extracellular or the part of host cell or membrane; metabolic processes such as primary and cellular metabolic processes including cyclic, heterocyclic and aromatic compound metabolism processes (data not shown).

Molecular function including binding (Score: 49) involves heterocyclic (Score: 49), organic (49), cyclic compound binding; small molecule (Score: 32) and nucleic acid binding activity (Score; 38) whereas and catalytic activity (Score: 26) include transferase activity (Score: 19) with nucleotide transferase activity (Score: 40).

### Homology modelling and structural validation of X- domain

**Figure 2:**
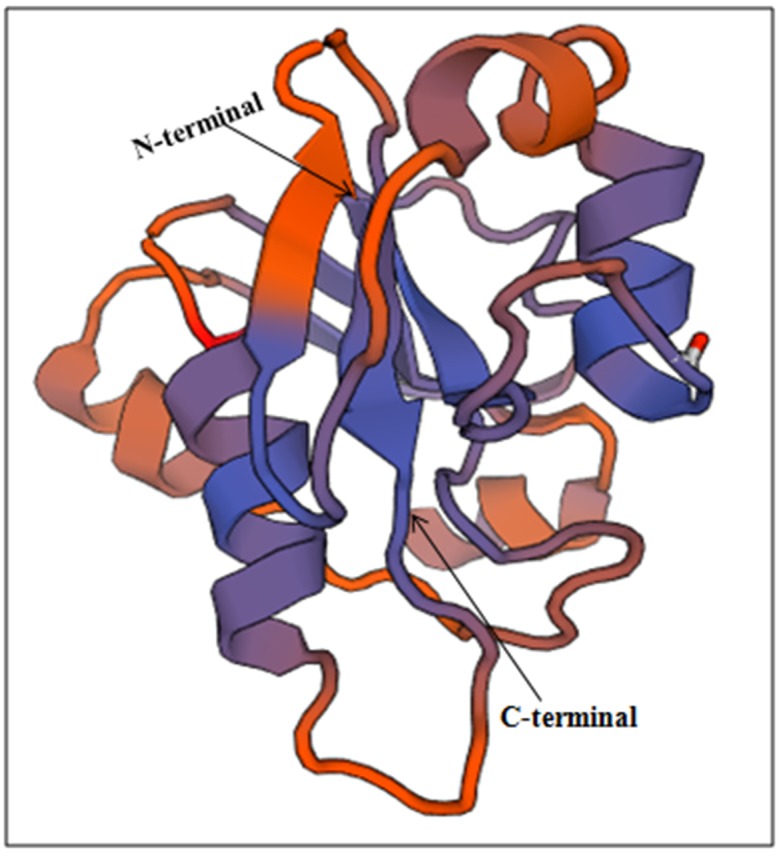
HEV X-domain structure with helix, strands and coil predicted by Swiss Model

X domain target sequence was inserted as input (fasta format) in Swiss-Model workspace. The Swiss-MODEL template library (SMTL) was searched with HHBlits [34] resulted in total 120 templates. Among the 5 most favourable template (1spv.1.A; 5cms.1.A; 519k.1.A, 5cb3.1.A and 2x47.1.A), 1spv.1.A target sequence was selected based on the Qualitative Model Energy Analysis (QMEAN) score (-2.86), Global model quality estimate (GMQE) 0.59, percentage of sequence identity (24.09), similarity (32%) and coverage (87%) (data not shown).Model was based on target-template alignment using ProMod3, where insertion, deletions remodelled and side chains were then rebuilt. Our model showed resemblance with Ispv.1 (putative phosphatase of E. coli) and identified as putative polyprotein/phosphatase. The model so generated was saved in PDB format (Figure 2). Further structure assessment was performed i.e Ramachandran plots (favoured 87.41%) and MolProbity score (2.34), clash score 16.66 at A18TRP-A94LEU; A75ARG-A76LEU; A18TRP-A59TYR, with cis non-proline (1/125) A124PRO-A125GLY, Twisted proline (1/11) A28ARG-A29PRO and beta deviations at A42PRO, A94 LEU and A77GLU (data not shown). This PDB file was validated by QMEAN analysis (https://swissmodel.expasy.org/qmean/project/dM3RTW/) [35] showed score of -2.86.

Stereochemical quality of the Swiss model predicted X-domain structure was evaluated by plotting Ramachandran map (PROCHECK). 85.1% of the total residues (137) were found in the core (A; B; L) whereas 13.2% of residues were in the allowed (a; b; l) regions. Disallowed region constitute of 1.8% of the residues. Good quality model of X domain was predicted by analyzing 118 structures of good resolution (2.0 Å) and R-factor (<20%). PROCHECK analysis showed max deviation of 21.0 (residue properties), with bond length/angle of 5.8 and 77.8% planar groups within the limits.

**Figure 3:**
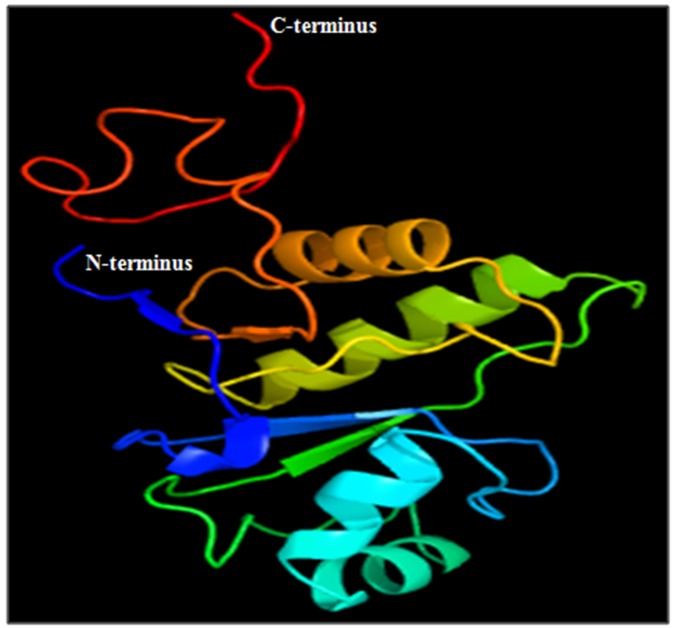
HEV X-domain structure predicted by Phyre2

Similarly, the homology modelling of X domain was performed by Phyre2.Based on the 6 templates (c5fsuA, c2x47A, c5iitC, c5kivA, c5fszA and d2acfa1), protein model was generated with 87% of the residues modelled at >90% confidence (Fig. 3) with coordinates (A): X: 51.738, Y: 33.604, Z: 42.515 (based on heuristics to maximise confidence, percentage identity and alignment coverage). Secondary structure prediction by Phyre2 was described as Disordered (13%), Alpha helix (36%), and beta strand (22%) (data not shown).

Phyre2 predicted structural model was evaluated for the stereochemical quality using Ramachandran map (PROCHECK). The 84.1% of the residues were found in the core (A; B; L) whereas 12.1% of residues were in the allowed (a; b; l) regions. However 2.3% residues were aligned in generously allowed region (~a, ~b, ~l), whereas disallowed region constituted 1.5% of the residues. Among residual properties max deviation was 4.1, bond length/angle 10.5 with 2 cis-peptides with 98.3% planar groups within limits.

**Figure 4:**
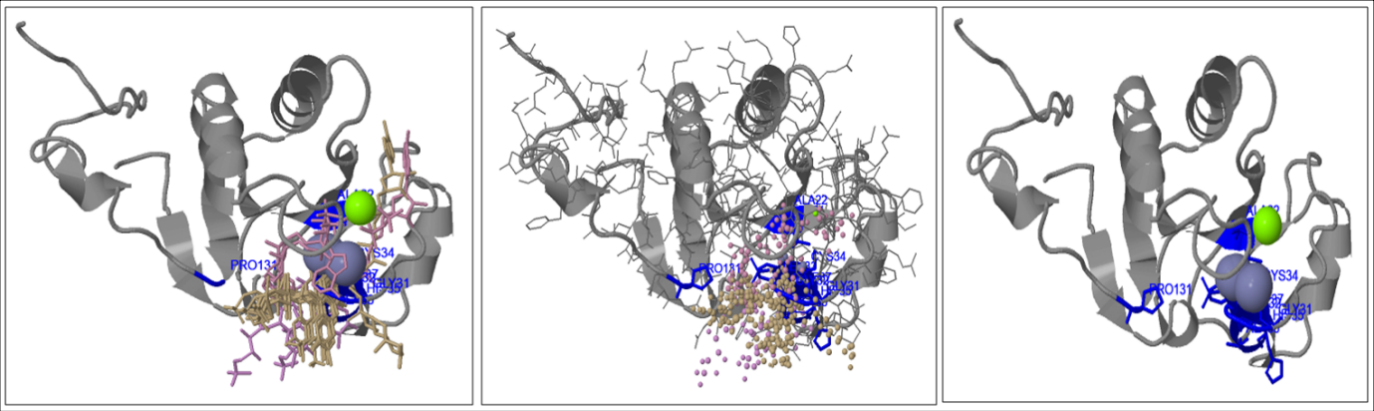
3-D structure of HEV X-domain with Mg2+ and Zn2+ ion ligands binding to active site (Phyre2)

The model was structured based on multi-template/ab initio with confidence score of 87. The 3D ligand site prediction in the final selected model was based on the cluster 1, showing 23 ligands and 18 structures. Total of 10 binding sites were predicted, at residue no. 21 (Asn: 12 contacts), 22 (Ala: 11 contacts), no. 31-37 (Gly: 15 contacts, Gly: 18 contacts, Leu: 21 contacts, Cys: 19 contacts, His: 11 contacts, Ala: 12 contacts, Phe: 13 contacts), no.131 (Pro: 10 contacts). Mg (1) and Zn (6) was predicted as metallic heterogen ligands, binding to the predicted ligand binding site of the modelled X domain (Figure 4).

## Conclusion

We report the structural model of HEV X domain with predicted active site for ligand binding. This provides insights into the functional role of X-domain in viral pathogenesis.

## Financial Support

VT is the recipient of ICMR JRF/SRF fellowship provided by Indian Council of Medical Education and Research (ICMR), New Delhi.

## Competing Interest

None

## Figures and Tables

**Table 1:** Physiochemical parameters computed using Expasy's ProtParam and SMS tool.

S. No.	Physio-chemical parameters	Values
1	No. of amino acid (aa)	158
2	Molecular weight (MW)	17433.62
3	Theoretical Isoelectric point (pI)	5.94, 6.34*
4	Aliphatic Index	70.57
5	Instability Index	37.18 (Stable)
6	Extinction Coefficient (All Cys form Cysteine)	28670
7	Extinction Coefficient (All Cys reduced)	28420
8	Total no. of negatively charged residues (Asp+Glu)	17
9	Total no. of positively charged residues (Arg+Lys)	14
10	GRAVY (Grand average of hydropathicity)	-0.278
*pI determined by SMS Version2		

**Table 2:** Secondary structure elements prediction by SOPMA

S. No.	Secondary structure elements	Values (%)
1	Alpha helix (Hh)	34.81%
2	310 helix (Gg)	0.00%
3	Pi helix (Ii)	0.00%
4	Beta bridge (Bb)	0.00%
5	Extended strand (Ee)	20.25%
6	Beta turn (Tt)	4.43%
7	Bend region (Ss)	0.00%
8	Random coil (Cc)	40.51%
9	Ambiguous states (?)	0.00%
